# Utah’s Family High Risk Program: Bridging the Gap Between Genomics and Public Health

**Published:** 2005-03-15

**Authors:** Jenny Johnson, Rebecca T Giles, LaDene Larsen, Joan Ware, Ted Adams, Steven C Hunt

**Affiliations:** Utah Department of Health Chronic Disease Genomics Program; Utah Department of Health, Salt Lake City, Utah; Utah Department of Health, Salt Lake City, Utah; Utah Department of Health, Salt Lake City, Utah; University of Utah, Cardiovascular Genetics Research Program, Salt Lake City, Utah; University of Utah, Cardiovascular Genetics Research Program, Salt Lake City, Utah

## Abstract

**Background:**

Family history is a simple yet powerful genomic tool that can identify individuals and entire populations at risk for diseases such as heart disease, cancer, and diabetes. Despite its use for predicting disease, family history has traditionally been underused in the public health setting.

**Context:**

A program for identifying families at risk for a variety of chronic diseases was implemented in Utah. Utah has population characteristics that are unique among the United States. Although the land area is large, most residents live within a relatively small geographic area. The religion of 70% of the residents encourages the recording of detailed family histories, and many families have access to records dating back to the 1800s.

**Methods:**

From 1983 through 1999, the Utah Department of Health, local health departments, school districts, the University of Utah, and the Baylor College of Medicine implemented and conducted the Family High Risk Program, which identified families at risk for chronic diseases using the Health Family Tree Questionnaire in Utah high schools.

**Consequences:**

The collection of family history is a cost-effective method for identifying and intervening with high-risk populations. More than 80% of eligible families consented to fully participate in the program. A total of 80,611 usable trees were collected. Of the 151,188 Utah families who participated, 8546 families identified as high-risk for disease(s) were offered follow-up interventions.

**Interpretation:**

The program was revolutionary in design and demonstrated that family history can bridge the gap between genetic advances and public health practice.

## Background

With the arrival of the genomics era, we are faced with the challenge of how to apply genetic knowledge to public health practice (1). A challenge of this magnitude also presents a great opportunity to more effectively target health promotion activities to individuals and families at highest risk. Family history holds promise as one of the keys to unlock this opportunity because it captures genetic and environmental components of diseases, including shared cultural and behavioral risks (1,2). However, despite the fact that family history plays a significant role in many chronic diseases of public health concern such as heart disease, asthma, cancer, and diabetes (3), it has traditionally been underused in the public health setting (2,3). Few examples of public health organizations that have used family history as a long-term, cost-effective tool for identifying and intervening with high-risk populations are documented in the current literature.

From 1983 through 1999, the Utah Department of Health (UDOH) partnered with local health departments, school districts, Baylor College of Medicine, and the University of Utah School of Medicine Cardiovascular Genetics Research Clinic (UCVG) to develop and implement the Family High Risk Program (FHRP). The FHRP used the Health Family Tree Questionnaire (HFT) to identify families at increased risk of developing major adult-onset diseases that could be prevented, delayed, or treated effectively with early interventions.

## Context

The late Roger R. Williams, MD, former director of UCVG and founder of Make Early Diagnosis to Prevent Early Death (MED PED) (4), was instrumental in developing the FHRP. Williams' research on familial trends in coronary-prone pedigrees showed that approximately 14% of the Utah population contributed to 72% of the state's total early coronary deaths (5). In light of these findings and other epidemiological studies (R. Williams, University of Utah, unpublished data, 1982), Williams joined efforts in 1980 with investigators at the Baylor College of Medicine to further develop the HFT as a tool to accurately collect and analyze familial disease tendencies (6).

There has been some criticism of the FHRP because it was implemented in a unique population compared with the total U.S. population. Although Utah has a large land area, the majority of the state's 2,351,467 (7) citizens live along the Wasatch Front, a stretch of land 90 miles long and 20 miles wide. In 2003, approximately 32% of Utah's population was aged 18 years and older (7), reflective of the increase in public school enrollment since the 1980s. The religious background of 70% (7) of Utah's citizens encourages the recording of detailed family histories, and access to genealogical records dating back to the 1800s is available for many Utahns in the Family History Library of the Church of Jesus Christ of Latter-day Saints (8). Family pedigrees in Utah are typically larger than in other states, and many families reside in the same area for multiple generations. Utahns have a favorable relationship with public health agencies and the state's major universities, which has enabled numerous population studies. Finally, researchers also have access to a variety of records from the Utah Population Database that aid in developing these studies (8).

Despite concerns that such unique characteristics would affect the program's ability to identify and intervene with high-risk populations in other states, data from Texas students showed similar results when compared with data from Utah students (5). These data warrant further exploration for using family history to bridge genetic advances and public health practice on a national scale.

## Methods

The original version of the HFT was developed to enhance risk-reduction messages in health education courses (9,10). Baylor investigators used the HFT in Texas from 1980 to 1986 with 6578 families from four multiethnic cities within the Waco Independent School District (5,6). The tool was used in the Waco Family Health Program, which was designed to increase students' knowledge of the risks and prevention of cardiovascular disease and to promote behavior changes. However, little testing was done on the validity of the HFT because of the original intent to use it as an educational tool. From 1982 to 1985, Williams received funding from the Thrasher Research Fund to further develop and assess the HFT in Utah high schools (5,6).

Partnerships among public health, community, and research-based entities played an important and unique role in the FHRP. Previous working relationships between Williams and the UDOH provided the infrastructure needed for program implementation. Key individuals from high schools, school districts, local health departments, hospitals, medical associations, and nonprofit agencies (e.g., American Cancer Society) were recruited to disseminate the FHRP throughout Utah. Voluntary training sessions were conducted with participating teachers and local health department personnel prior to program implementation. During the sessions, teachers received curriculum materials, optical scanner forms, and HFTs for their students at no cost to themselves or the school districts. Training sessions were also available for health care providers working with high-risk families. Continuing medical education was available through grand rounds, a self-study course, and a set of videos. 

**Figure 1:**
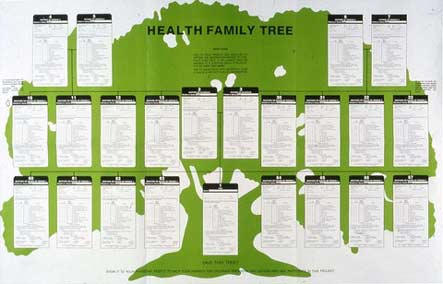
The Health Family Tree questionnaire collected family medical history from students enrolled in required high school health education courses in Utah from 1983 through 1999. Reprinted with permission from Elsevier (9).

**Figure 2:**
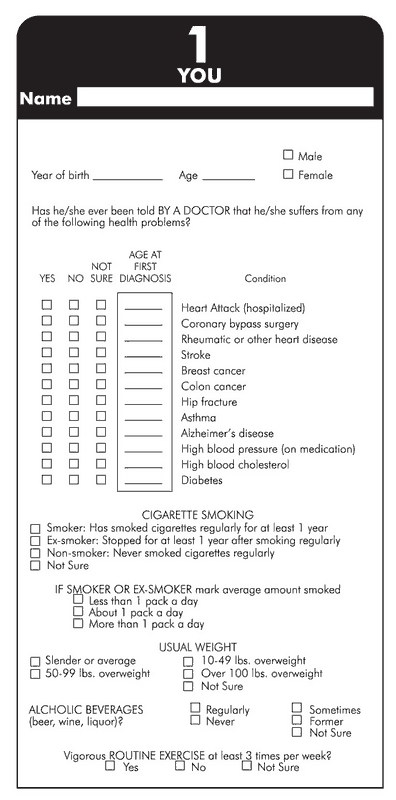
Information collected for the Health Family Tree questionnaire included age of disease onset for a number of chronic diseases as well as lifestyle risk factors for each family member of participating students. Reprinted with permission from Elsevier (9).

The program was pilot tested in 1983 with more than 1000 students in seven high schools, far exceeding expectations. Revisions to program materials were then made, and full program implementation began in fall 1983. Material development was supported by the U.S. Department of Health and Human Services through the Centers for Disease Control and Prevention (CDC), National Heart, Lung, and Blood Institute (NHLBI), and Utah State general funds. The HFT was designed to collect three generations of family medical history (Figure 1); its large format (two feet by three feet) was designed to fit comfortably on a kitchen or dining room table to encourage family participation. The information included lifestyle factors and certain disease conditions (Figure 2) for siblings, parents, aunts and uncles, and grandparents of students enrolled in required high school health education classes. In 1995, hip fractures, asthma, and Alzheimer's disease were added to the HFT. The condition "other cancers" was removed in 1996.

Teachers used the HFT as the focus of a four-part curriculum (Table) on the prevention of common chronic diseases (11). A curriculum guide was written and updated periodically by FHRP staff with input from participating teachers. Students were encouraged to complete the HFT assignment whether or not they were a blood relative to their family members, and parents were required to give consent for their student to participate before data collection. Three participation options were available for selection. Option one gave students consent for full participation in the program. This included an evaluation of the HFT, permission for the UDOH, local health department, or UCVG representatives to contact the family, and permission to store names, addresses, and phone numbers in confidential research files at UCVG for further research. Option two allowed for partial participation that included permission for the student to complete the HFT but receive no evaluation, follow-up visits, or further contact. However, data from the HFT were stored anonymously at UCVG. Option three was nonparticipation in the program, and students were given alternate assignments to complete. Nonparticipation had no effect on the student's grade as long as alternate assignments were completed. 

After collecting information for the HFT, students transferred the data onto optical scanner forms and completed a demographic survey. This allowed UCVG researchers to efficiently analyze the information and determine the disease risk for each family, or Family History Score (5,6,9,12). Statistical analyses of family risk were calculated separately for each parent's family, excluding adopted relatives, which helped identify high-risk parental pedigrees even if the student was not a blood relative (6). Computer-generated reports summarizing risk of disease(s) and behavior-change recommendations to reduce risk were mailed by UCVG to families who consented to provide contact information. A list of high-risk families was also sent to the UDOH.

**Figure 3:**
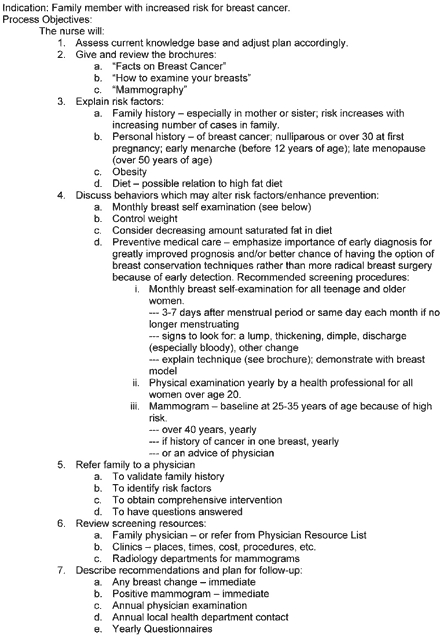
Standards of care for breast cancer used by local health departments and public health nurses during follow-up care of high-risk families. Reproduced with permission from Oncology Nursing Society (11).

Family-based interventions were offered to families identified by the HFTs as high-risk for a particular disease(s). Williams and the UDOH developed nursing protocols and standards of care (Figure 3) to ensure appropriate and consistent care was provided to all high-risk families. The UDOH coordinated with public health nurses at local health departments in the community where the family lived to provide personalized medical assessments, education, and referrals to health care providers during in-home visits. Behavior-change classes (e.g., smoking cessation, cooking classes), free medical screenings (e.g., blood pressure, cholesterol), and educational resources (e.g., handout on "Questions You Might Want to Ask Your Physician About," Family Health Record Book) (11) were also available to high-risk families.

The in-home visits were highly effective during the early years of the program because interventions assessed the entire family structure, taking into account not only medical history but also social structure, lifestyle behaviors, and family dynamics. In-home visits allowed nurses to provide individualized care for each family as well as emotional support as they developed healthier behaviors. However, as funding and time constraints were placed on the UDOH and local health departments, fewer families received the care that program planners had intended. Changing family dynamics throughout the period of the FHRP, such as fewer two-parent households and more women working outside the home, proved to be difficult barriers and reduced the effectiveness of interventions. 

Evaluations on intervention effectiveness were conducted over a period of ten years with a cohort of high- and average-risk families. FHRP staff also conducted periodic satisfaction surveys with teachers, students, public health nurses, and high-risk families throughout the program to guide program activities.

## Consequences

The FHRP demonstrated that the collection of family history is a cost-effective method for identifying and intervening with high-risk populations. Strategies for reducing program costs were identified by UCVG early in program development. By designing optical scanner forms for data input, the time and expense required for analysis decreased dramatically. Costs were reduced from $25 per analysis to less than $10 per analysis for students who completed the HFT but did not receive follow-up interventions (5,6,9). Cost for each high-risk family that received interventions was approximately $27 (5). This cost included data processing, report generation and mailing, and in-kind donations from UDOH and local health departments. Costs for both high- and average-risk families compared favorably with other types of behavior-modifying programs at that time.

Although the UDOH terminated the program in 1999 because of a lack of funding, data from HFTs were collected by UCVG until 2002. A total of 80,611 usable trees were collected from students during the 20-year span. More than 80% of eligible families consented to fully participate in the program (option one). Twelve percent of eligible families consented to partially participate (option two), and only 7% refused to participate (option three) (T. Adams, unpublished data, 2004). Families who refused to participate during later years of the program often did so because older children had already completed an HFT and, if their family was at high risk, they had already been offered follow-up care from local health departments. Teacher participation in the FHRP was also high, with approximately 284 teachers from 55 high schools voluntarily participating, many for the entire length of the program.

Of the 151,188 Utah families who participated in the program, 17,064 were identified as high-risk for coronary heart disease and 13,106 were identified as high-risk for stroke (5), many of which might have otherwise remained undiagnosed by both their health care providers and the public health system. The UDOH offered interventions to 8546 high-risk families. During the early years of the FHRP, an average of 90% of high-risk families had some form of follow-up contact; more than 60% of the contacts were in-home visits (J Ware, oral communication, January 2004). Focus groups and telephone surveys conducted with high-risk families showed that the majority of participants were grateful to be told about their disease risk, and 95% of parents felt the project was a valuable learning experience for their child. Families were motivated to make long-term behavior changes simply by knowing their family history, and families showed compliance with targeted health promotion messages. Preliminary review of the 10-year evaluations showed that both high- and average-risk families reported an increase in healthy lifestyle behaviors, such as obtaining yearly medical exams and blood pressure checks, as a direct result of participating in the FHRP. A higher increase in healthy lifestyle behaviors was seen in families that received interventions (Utah Department of Health Chronic Disease Genomics Program, unpublished data, April 2004). Complete analysis of intervention effectiveness is underway and will be published in a subsequent article.

The long-term success of the program has generated worldwide interest. Countries interested in using the program included Canada, France, Germany, Hungary, Japan, Russia, South Africa, and Sweden. FHRP staff received additional contacts from universities and state health departments in California, Florida, Iowa, Maine, Minnesota, New Jersey, North Carolina, Oregon, and Texas. In 1986, the FHRP was recognized as a "distinguished community health promotion program," receiving the U.S. Department of Health and Human Services Secretary of Health's National Award of Excellence. The FHRP was also a semifinalist in 1986, 1988, and 1990 in the Innovations in State and Local Government Awards, an awards program of the Ford Foundation and the John F. Kennedy School of Government at Harvard University.

## Interpretation

As we enter the genomics era, family history will become an increasingly important tool for bridging genetics and disease prevention strategies. The FHRP provides a practical example of what geneticists have long known — that family history can be used to predict disease susceptibility in high-risk individuals, and these individuals can benefit from personalized interventions, thus reducing their risk of disease (2,13,14). In a recent article, Guttmacher et al reiterated the importance of applying this "free, well-proven, personalized genomic tool" in preventive medicine (13). We believe the FHRP successfully demonstrates that family history, used as a simple genomic tool, can bridge the gap between genetic knowledge and public health practice and can serve as a reminder of the importance of utilizing family history information in disease management and prevention (13,14). Use of family history has great potential to educate and motivate individuals to comply with preventive health strategies; this potential is suggested in the literature (15) and by preliminary review of FHRP data.

Experience from the FHRP has provided a springboard for activities in public health, including within the CDC Office of Genomics and Disease Prevention (OGDP), to explore the usefulness of genomics in public health (2,15). The OGDP launched an initiative in 2002 to understand how family history can be used in health promotion and disease prevention and has begun to develop a family history tool that can be used within a strategy to integrate genomics into public health practice (1,2). The U.S. Surgeon General launched a National Family History Initiative to encourage the public to use family history in their health care (13). Data and experience from the program have also enabled further medical genealogical research in the MED PED program (4) and NHLBI Family Heart Study (5).

We believe the long-term success of the FHRP demonstrates that family history can enhance traditional health promotion and disease prevention strategies in public health, community, and health care settings. Health professionals must discover the value of genomics (13,14) by exploring the development of programs similar to the FHRP and integrating recommendations from current family history research into their own practice. Perhaps outcomes of these projects will again prove what we have already learned from the FHRP — that family history holds the key for applying genomics to today's public health concerns.

## Figures and Tables

**Table:** Learning Objectives of the Family High Risk Program, Health Family Tree Curriculum for High School Health Education Programs in Utah, 1983–1999

**Part 1** Heredity and Your Health	The student will be able to: Recognize definitions of various chronic diseases. Explain basic principles of heredity. Explain the difference between a medical pedigree and standard pedigree or family tree. Define familial tendency. Discuss importance of identifying individuals with familial tendencies for disease.
**Part 2** Filling Out the Health Family Tree	The student will be able to: Complete the “You” section of the Health Family Tree pedigree form in class. Complete the Health Family Tree pedigree form at home with parental assistance.
**Part 3** Healthy Lifestyles	The student will be able to: Recognize that families with familial tendencies should have a physician’s supervision to reduce risk. Recognize that there are controllable risk factors that impact those with familial tendency as well as those without familial tendency. Describe the healthy lifestyle choices that will enhance the quality of life and decrease risk of chronic diseases.
**Part 4** Checking the Computer Scanner Forms	The student will be able to: Accurately edit the Health Family Tree data on the computer scanner sheets. Summarize the number of relatives who died of heart attacks, strokes, cancer, and diabetes, noting the age when they died. Looking at this summary, the students should decide if there could be a familial tendency.
